# Arthroscopic removal of a plastic soft drink bottle cap in the knee: a case report

**DOI:** 10.1186/1757-1626-3-72

**Published:** 2010-02-24

**Authors:** Simon Boyle, Joseph C Talbot, Quamar Bismil, Ernest Schilders

**Affiliations:** 1 Bradford Royal Infirmary, Duckworth Lane, Bradford, BD9 6RJ, UK

## Abstract

We report a rare case of late knee locking after an open knee injury in a polytrauma patient with a pelvic fracture and a contralateral femoral artery injury. Once the life and limb threatening injuries were addressed, debridement and washout of the knee wound was performed. X-rays and subsequent CT revealed only an undisplaced patella fracture. The patient presented 6 months later to a knee surgeon with recurrent locking. An arthroscopy was performed and a 10 mm plastic soft drink bottle cap was retrieved leading to the immediate resolution of symptoms without complications.

Open knee injuries require thorough debridement washout and joint assessment. Late locking should raise the suspicion of an intra-articular loose or foreign body. Arthroscopy is an excellent first line tool in the diagnosis and late management of this unusual problem.

## Introduction

Mechanical locking symptoms in the knee are a common presenting complaint to arthroscopic knee surgeons. These are often due to meniscal tears occurring after sporting injuries or secondary to age related degenerate change in the knee. Other intra-articular causes include loose osteochondral fragments, torn ligaments (eg. ACL), patellofemoral disorders and synovial plica. Much less commonly, knee locking can be due to the presence of a foreign body. This is almost always accompanied by a clear history of penetrating trauma to the knee, and this combined with radiographs often identifies the nature and location of the foreign body.

We report a case of a plastic bottle cap causing late knee locking after an open knee injury which was successfully identified and removed by arthroscopy.

## Case presentation

A 16 year old boy was involved in a motorcycle RTA sustaining an APC type I injury to his pelvis and injuries to both lower limbs. A 10 cm laceration was found over his left adductor canal associated with a pulseless left leg and a 5 cm laceration over his right knee laterally.

After immediate resuscitation he was taken to theatre where he underwent debridement, washout and end to end contralateral long saphenous vein grafting to a severed femoral artery to his left leg. Left thigh fasciotomies were performed and a debridement and washout was then performed on his right knee laceration where a 5 cm retinacular tear was noted. A second look, further debridement and washout was performed at 48 hours and the right knee wound closed. At 7 days post injury split skin grafts were applied to the fasciotomy wounds.

Plain x-rays and a CT of the right knee revealed an undisplaced patella fracture. This was immobilised, and physiotherapy and rehabilitation began at 4 weeks post injury. He made an uneventful recovery from his pelvic and vascular injuries and was mobilising fully at 3 months.

**Figure 1:**
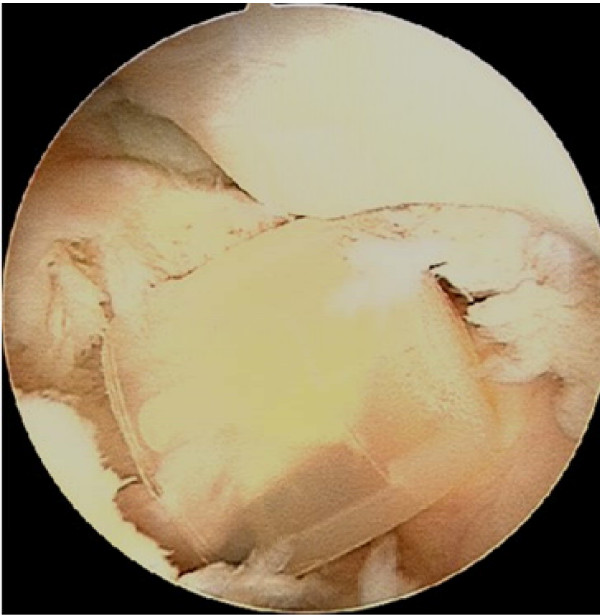
**Soft drink bottle cap within the femoral intercondylar notch, anterior to the ACL**.

He subsequently presented to a knee surgeon 6 months later with a history of mechanical locking in his right knee. An arthroscopy was performed using standard portals and a 10 mm soft drink bottle cap was found in the intercondylar notch anterior to the ACL (Figure 1). This was removed through the medial portal. The rest of the joint was normal with no osteochondral defects and the patella fracture had healed. He made a full recovery with complete resolution of his locking symptoms.

## Discussion

Foreign bodies in the knee from penetrating trauma have previously been removed successfully using arthroscopic techniques. These have included bullets [1,2], pencil lead [3], metallic stone [4] and metal debris [5-7]. We are unaware of any other cases of the successful arthroscopic removal of a plastic foreign body after an open knee injury presenting with late locking.

Polytrauma patients frequently present with significant life and limb threatening injuries which need immediate surgical intervention. Once these injuries are addressed it is important to correctly manage the other seemingly less significant injuries appropriately. This means that open joint injuries should undergo an immediate and thorough debridement and washout. This is also the best time to make a direct assessment of the intra-articular structures for signs of osteochondral damage, ligament disruption and foreign bodies. A second look at 48 hours should also aim to reassess the joint and act as a guide as to the need for further imaging.

Plain x-rays and CT imaging are excellent in demonstrating bony disruption however neither modality is good for demonstrating plastic foreign bodies. MRI will show plastic foreign material but if the presence of this or any intra-articular soft tissue disruption is not suspected, then this modality is often overlooked. This is particularly true in units with limited access to MRI scanners.

Arthroscopy offers an excellent combined diagnostic and therapeutic technique for managing a late locking knee after an open joint injury. This was performed as a day case procedure with a rapid return to normal activities and no complication was noted. We would recommend this technique as a first line in cases of late knee locking post open injury.

## Conclusion

Late locking after a previous open knee injury despite normal radiological investigations should raise the suspicion of an intra-articular foreign body. Clinical suspicion as to the presence of potential radiolucent foreign bodies should be raised. First line management should involve a thorough debridement washout and joint examination once life and limb threatening injuries have been stabilised. Arthroscopy is an excellent first line tool in the diagnosis and immediate management of this unusual problem.

## Consent

Written informed consent was obtained from the patient for publication of this case report and accompanying images. A copy of the written consent is available for review by the Editor-in-Chief of this journal.

## Competing interests

The authors declare that they have no competing interests.

## Authors' contributions

SB, JCT, QB conceived the case report and were major contributors in writing the manuscript. ES performed the surgery. All authors read and approved the final manuscript.
